# Morquio B disease: a case report

**DOI:** 10.3389/fped.2024.1285414

**Published:** 2024-03-04

**Authors:** Tara Gholamian, Harpreet Chhina, Sylvia Stockler, Anthony Cooper

**Affiliations:** ^1^ Faculty of Medicine, University of Ottawa, Ottawa, ON, Canada; ^2^ Department of Orthopedics, Faculty of Medicine, University of British Columbia, Vancouver, BC, Canada; ^3^ Department of Biochemical Diseases, University of British Columbia, Vancouver, BC, Canada; ^4^ Department of Biochemical Diseases, BC Children’s Hospital, Vancouver, BC, Canada; ^5^ Department of Orthopedic Surgery, BC Children’s Hospital, Vancouver, BC, Canada

**Keywords:** Morquio B disease, orthopedics, case report, MBD, mucopolysaccharidosis IV type B

## Abstract

Mucopolysaccharidosis IV type B, or Morquio B disease (MBD), is an autosomal recessive disorder caused by a genetic mutation in GLB1 gene encoding for β-galactosidase on chromosome 3p22.33. β-galactosidase deficiency can result in two different conditions, GM1 gangliosidosis and MBD, of which MBD has a milder phenotype and presents later in life with keratan sulfate accumulation in the retina and cartilage. In this case report, we present a patient diagnosed with MBD at the age of 5 after initially presenting with Morquio dysostosis multiplex and characteristic radiographic findings. Genetic testing confirmed that the patient has β-galactosidase deficiency due to mutation W273l/N484K on GLB1 gene. The patient exhibited elevated mucopolysaccharide levels in urine at 18 mg/mmol and demonstrated an abnormal band pattern of urine oligosaccharides on electrophoresis. The activity of β-galactosidase in his white blood cells was reduced to 12.3 nmol/h/mg protein. At the time of diagnosis, the patient did not present with gait and ambulation issues, but his ability to walk progressively deteriorated in his adolescence as a result of instability and pain in the ankle, knee, and hip joints, accompanied by a global decrease in muscle strength. This case report is the first in the literature to provide an in-depth exploration of the orthopedic treatment and follow-up received by a young adolescent with MBD to provide symptom relief and improve walking ability.

## 1 Introduction

Mucopolysaccharidosis IV type B, or Morquio B disease (MBD), is a rare autosomal recessive lysosomal storage disorder that occurs due to mutations in the genes encoding for β-galactosidase enzyme (1, 2). Deficiency in the enzymatic activity of β-galactosidase causes abnormal accumulation of glycosaminoglycans such as keratan sulfate (1, 2). This buildup can occur in the cornea, urine, and cartilage, where it causes abnormal endochondral ossification and bone growth.

MBD patients are diagnosed within the first few years of life due to their severe skeletal abnormalities. These abnormalities, termed dysostosis multiplex, of MBD include a disproportionate short stature, atlantoaxial instability, odontoid hypoplasia, platyspondyly, kyphoscoliosis, pectus carinatum, coxa valga, and genu valgum (3–5). Other manifestations include lax joints, trigger fingers, corneal clouding, sensorineural hearing loss, dental abnormalities, and cardiopulmonary complications (3, 4). MBD patients lack symptoms suggestive of Central Nervous System (CNS) involvement or storage disease in neuronal tissues, and they present with normal intelligence (3, 4). Orthopedic treatment provides symptomatic relief; however, it does not halt disease progression, leading to continual skeletal issues and pain for patients. MBD patients are at higher risk for death due to complications arising from valvular heart disease and respiratory failure, as well as spinal cord injury due to spinal cord compression and vertebral instability (1, 2).

The incidence of MBD ranges from 1 per 250,000 to 1,000,000 births and can vary by geography (6, 7). Despite the devastating health consequences and lowered quality of life experienced by these patients, there are few studies reporting on the natural history and treatments for patients with MBD. In this case report, we outline all aspects of care received by a patient with MBD. In addition, we hope to improve the care received by MBD patients by providing the first case report outlining the in-depth orthopedic treatment received by patients with MBD in their childhood and adolescence.

## 2 Patient information

### 2.1 Birth

The Caucasian male patient was born to non-consanguineous parents at 36.5 weeks via cesarean section following a normal pregnancy. He weighed 8 pounds and 3 ounces (50th percentile) at birth.

## 3 Clinical findings and diagnostic assessment

**Figure 1:**
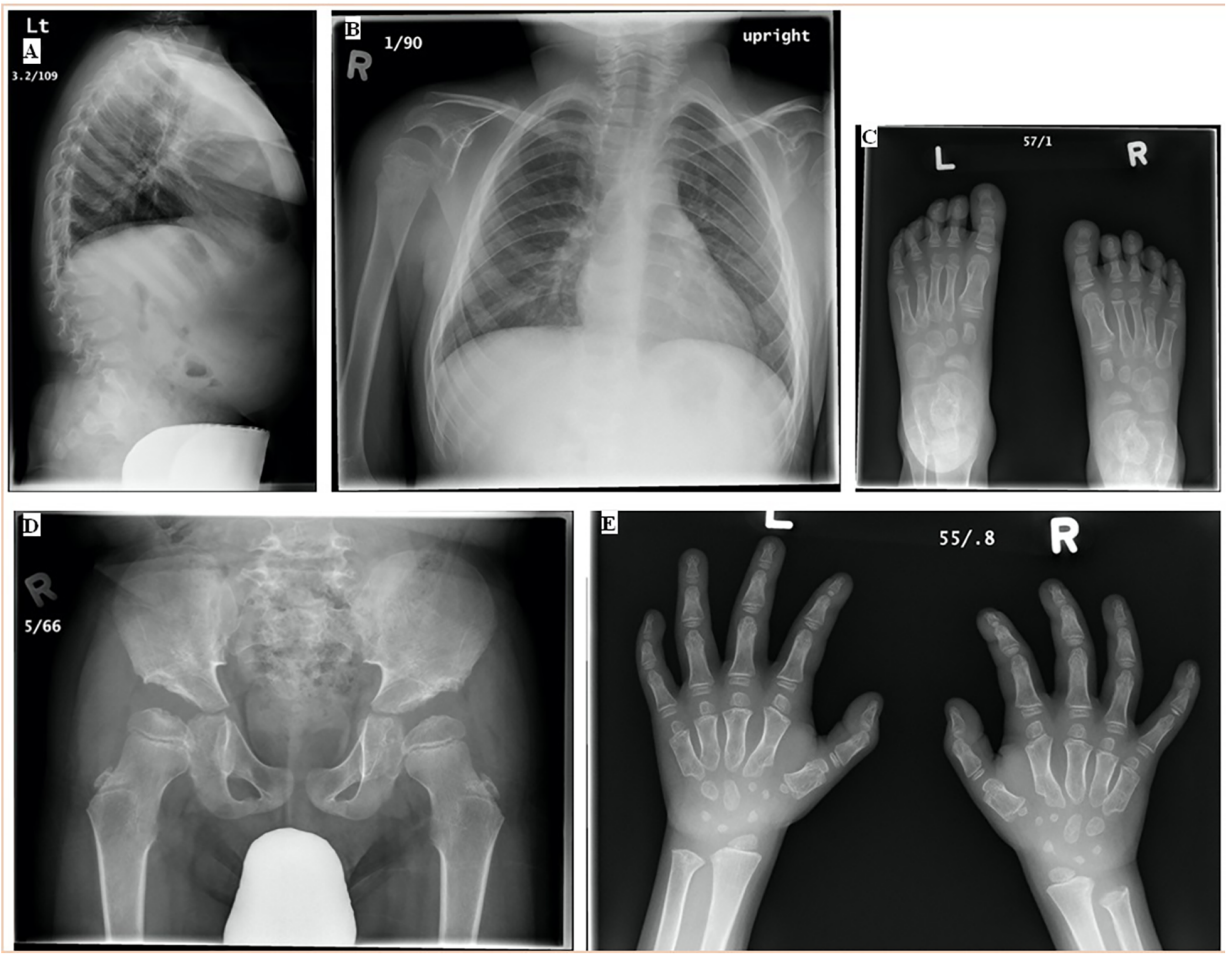
(**A**) Standing LAT and (**B**) PA spine, (**C**) AP bilateral feet, (**D**) AP hip, and (**E**) AP bilateral hands at diagnosis (7 April 2009 and 9 April 2009, 5 years). The spine had anterolisthesis of L1 and L2 with some anterior breaking through the lumbar spine. The skeleton was somewhat coarsely trabeculated. The pelvis had abnormal acetabular screws with tombstone appearing iliac bone, with some femoral head epiphyses irregularity. The ribs were slightly wide. The bones of the feet were osteopenic. The contour of all the tarsal bones and the metatarsal epiphyses was irregular. No focal bony abnormality was seen in the feet. There was proximal tapering of the second through fifth metacarpals bilaterally. The first metacarpal was short, and there was a well-defined notch in the radial aspect of the first metacarpal head bilaterally. There were slight irregularities in the phalangeal epiphyses. The carpal bones were small and slightly irregular in contour. There was bilateral negative ulnar variance, more marked on the right than left. LAT, lateral, PA, posterior-anterior, AP, anterior-posterior.

The patient was diagnosed with MBD at 5 years of age after being examined by a physiotherapist due to weak lower extremities. The patient reported trigger fingers, tight hamstrings, and proximal muscle weakness in his lower extremities that caused him difficulties with squatting, balancing, and walking with a stable gait. The patient was 107.9 cm tall (25th–50th percentile) and weighed 22.1 kg (75th–90th percentile), with a head circumference of 52.8 cm. Physical features suggestive of MBD included short stature, a barrel-shaped chest, pectus carinatum, short neck and trunk, longer limbs, hyperextensible joints, and thoracic kyphosis. He was described at that time as having mildly coarse facial features along with a flat midface. Radiographic features included flat vertebrae, a hypoplastic odontoid with a stable relationship to C1/C2, thoracic kyphosis, lumbar hyperlordosis, proximal tapering and shortened metacarpal bones, small and irregular carpal and tarsal bones, platyspondyly, hip dysplasia, and a flared lower coastal margin (Figure 1). The patient exhibited elevated mucopolysaccharide levels in his urine. On electrophoresis, an abnormal band pattern of urine oligosaccharides was found, resembling a GM1 gangliosidosis control. The activity of β-galactosidase in his white blood cells was shown to be reduced, measuring at 12.3 nmol/h/mg protein, in an enzyme assay. Molecular testing confirmed the patient to be compound heterozygous for two pathological mutations in GBL1 gene (W273l/N484K).

Baseline MRI (9 years of age) showed thoracic kyphosis measuring 4° and narrowing of the spinal canal diameter. Diffuse lumbar spinal stenosis was observed, as the canal diameter was reduced due to prominent disc annuli with associated nerve root crowding. The exit foramina of all lumbar nerve roots were narrow, especially on the left side. The dens were hypoplastic, with a stable atlantoaxial distance. Anterior beaking of the C3–T2 vertebral bodies was observed, leading to compression of the anterior superior and inferior endplates.

At the age of 10 years, the patient was 123.0 cm tall (70th percentile, −2.5 SD for age) and weighed 30.4 kg (38th percentile, −0.3 SD for age), with a head circumference of 53.5 (52nd percentile, +0.0SD for age). A bone density analysis showed low bone mass for his age. The ankle valgus measured 32° bilaterally. The patient reported that he could walk independently for long distances (+1 km) slowly with the aid of custom bilateral UCBL in-shoe orthotics but preferred using a wheelchair.

## 4 Therapeutic intervention

### 4.1 Childhood (2–12 years): orthopedic visits

At 11 years, the patient underwent bilateral screw hemiepiphysiodesis of the distal tibia to correct ankle valgus and prevent further bone deformation and cartilage damage in the talar dome. An upper extremity orthopedic surgeon was consulted regarding his bilateral triggering fingers. The digits affected included the right thumb, right index finger, right long digits, and left ring digit. Although the patient did not report pain, functional impairment due to locking was observed, which he could manually correct. In addition, despite relative ulnar shortening compared to the radius, the patient exhibited good wrist strength with normal pronation and supination, leading to the conclusion that corrective surgery was not needed at the time.

**Figure 2:**
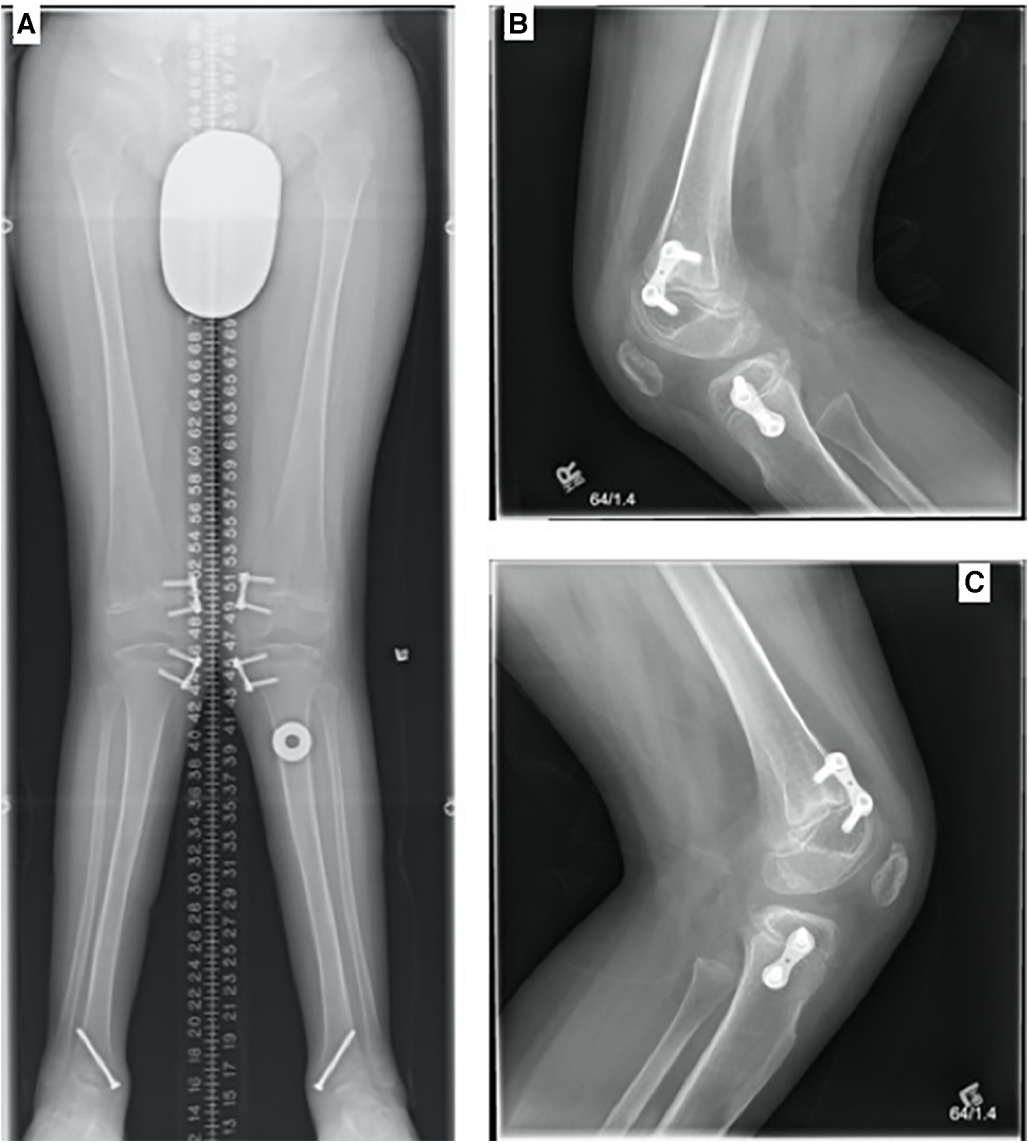
(**A**) Standing AP full leg length, (**B**) LAT right knee, and (**C**) LAT left knee (28 February 2016, 12 years). Bilateral genu valgum was present, with bilateral eight plates and screws transfixing the medial aspect of the distal femurs and proximal tibia bilaterally. The hardware was intact and unchanged in position. Single screws were medially transfixing the distal tibia bilaterally. No significant leg length discrepancy was present. Radiographic characteristics of MBD such as irregular articulating surfaces of the epiphyses were noted, worst at the talar domes.

At the age of 11 years, the progressing genu valgum was treated by bilateral distal femoral and proximal tibial medial hemiepiphysiodesis using a tension band plate. The patient achieved 125° of flexion bilaterally, with a right flexion contracture of 5° and a left contracture of 15°, for which physiotherapy was prescribed. At 3 months post-surgery, the patient reported decreased conditioning, as he had been walking about half of his previous distance due to fatigue. Examination revealed that the right knee fully extended to 0°, while his left knee extended to −5° with a soft endpoint. Also, there existed 10° of valgus bilaterally. By 8 months, the patient reported greater ease in walking, with a self-reported impression of improved alignment. The distal medial screw on the right side had aligned with the right heel in neutral with a slight varus alignment; consequently, the screw was removed when the patient reached the age of 12 years (Figure 2). While there was significant improvement in his overall alignment, he was reluctant to walk further due to discomfort around his hip abductors, associated with weakness and easy fatigability. On the right side, he achieved almost neutral alignment with full knee extension, while on the left side, he presented with a 10° knee flexion contracture.

**Figure 3:**
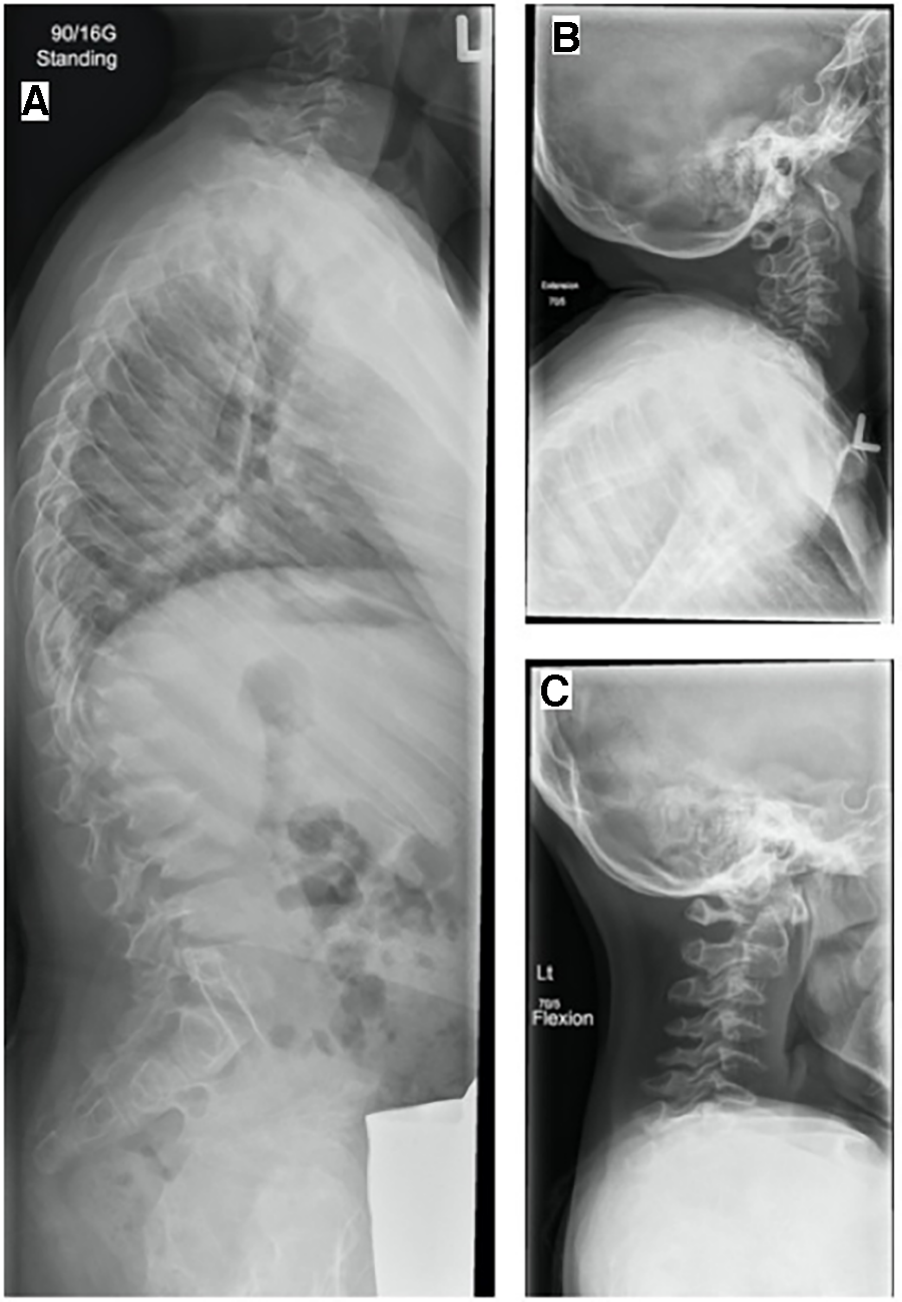
(**A**) Standing LAT spine, LAT cervical spine, (**B**) extension, and (**C**) flexion (7 May 2015, 12 years). Radiographs demonstrate vertebra plana, hypoplastic dens, and marked vertebral body beaking in the upper lumbar spine characteristic of Morquio disease. A broad-based kyphosis remained centered on the thoracolumbar junction, with anterior subluxation of T12 with respect to L1.

At 12 years of age, the patient exhibited a full range of motion in the neck. Even though a hypoplastic dens was observed on MRI scans and radiographs, no significant instability was evident radiographically or clinically. Radiographs showed a broad-based kyphosis centered on the thoracolumbar junction, in addition to anterior subluxation of T12 with respect to L1 (Figure 3).

### 4.2 Childhood (2–12 years): various department visits

At the age of 5 years, mild corneal clouding was noted by an ophthalmologist. A polysomnogram, audiology visit, assessment of cognition, and nerve conduction study yielded normal results. At the age of 7 years, the patient underwent surgical repair for a right-sided inguinal hernia.

A soft systolic ejection murmur was detected at the mid-left sternal border, with normal aortic and mitral valves and flow. At the age of 6, an echocardiogram (ECHO) revealed redundant cords to the anterior mitral leaflet with leaflet thickening without regurgitation. At the age of 8 years, a grade 2/6 pansystolic murmur was heard at the apex, radiating to the left sternal border, indicating mitral regurgitation. The patient reported not being physically restricted by his cardiac function.

Follow-up with the biochemical diseases team at the senior author's institution identified the patient’s short stature, as his height at the age of 5 was at the 25th percentile, but at the age of 10, he measured −8 cm below the 3rd percentile. At the age of 12, the patient measured 127.8 cm and weighed 38.5 kg.

## 5 Follow-up and outcomes

### 5.1 Adolescence (13–21 years): orthopedic visits

At the age of 13, the patient reported experiencing hip pain at night. On examination, he exhibited mild Trendelenburg positivity (more on the left side than the right). Alignment was corrected on the right side, but it was still persistently valgus on the left. The patient demonstrated full 5/5 motor function in the lower extremities in myotomes L2 through S1. In the decubitus position for specific testing of the hip abductors, they were found to be weak, with a strength of two out of five.

At the age of 13, the metaphyseal screws in the right distal femur, right proximal tibia, and left ankle were removed. The bilateral knee flexion contractures persisted at 10° on the left and 5° on the right.

Five months post-operation, the patient reported that his contractures and gait had improved with hydrotherapy and physiotherapy. No gross genu valgum was present, but the flexion contracture persisted between 0° and 5° bilaterally. However, due to radiographic evidence of recurring genu valgum on the right, the plan was to re-insert a nail on the right side 8-plate. By 8 months post-operation, the left knee had slightly overcorrected, which was desired due to him being a high-risk candidate for a potential rebound. The patient was able to stand well with neutral alignment on the right and slight varus on the left. He exhibited full range of motion (ROM) to his knees bilaterally, with significant right-sided hamstring tightness.

**Figure 4:**
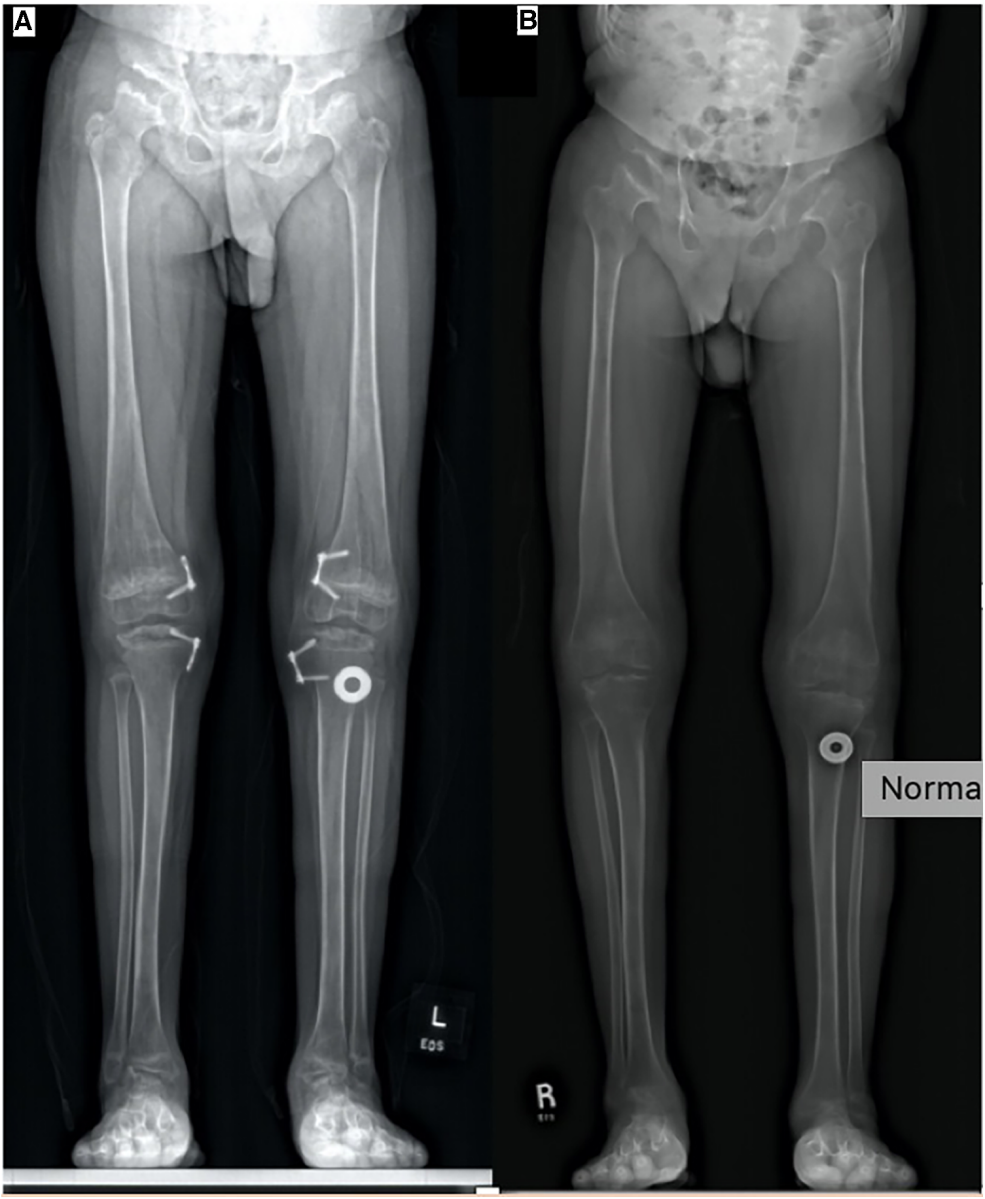
(**A**) Standing AP full leg length (16 March 2018, 14 years). Tension band plates and screws on the medial aspect of the right and left distal femur and proximal tibia can be seen, and right-sided plates only have one transfixing epiphyseal screw. Left genu valgum improved. Epiphyseal flattening and irregularity involving bilateral femoral heads and talus can be observed. (**B**) Standing AP full leg length (8 March 2021, 17 years). Tension band plates and screws have been removed from the medial aspect of the right and left distal femur and proximal right tibia. Alignment of the lower extremities has improved without genu varum or valgum, with osseous features of MBD remaining unchanged.

At the age of 14, he underwent removal of hardware from his left distal femur and left proximal tibia. Radiographs confirmed varus alignment of the left leg with recurrent valgus on the right localized to the proximal tibia (Figure 4). At the age of 15, he had re-insertion of 8-plate metaphyseal screws to his right distal femur and right proximal tibia. Post-re-insertion, he denied pain but reported being in his wheelchair predominately with minimal exercise tolerance. It is difficult to know whether this was purely due to mechanical inability or if other factors such as motivation played a role. The knees were a few degrees shy of full extension. On the radiograph, his right leg was nearly corrected, and growth plates were still open.

At the age of 16, the patient denied any hip, knee, or wrist pain. He had achieved adequate correction and was sitting at neutral. The hardware from the right and left distal femur and right proximal tibia were removed. At the 6-month follow-up, the patient had a Trendelenburg gait, with bilateral flexion contractures measuring 5°. His left foot had a slightly collapsed arch compared to the right, for which he completed posterior tibial tendon exercises and wore shoe inserts. His walking tolerance was 100–150 m.

The patient reported no pain or discomfort in his cervical, thoracic, or lumbar spine and no subjective sensory or strength deficits in his extremities. He had weak hands and intrinsics, with finger escape observed during sustained finger adduction. Even though he had known odontoid hypoplasia, there was no cervical spine instability on flexion or extension. His kyphotic deformity was stable.

### 5.2 Adolescence (13–21 years): various department visits

At the cardiology follow-up at 13 years of age, there was a 10%–15% increase in left ventricle outflow tract obstruction due to the mitral valve leaflet partially bulging into the tract. The patient reported sleeping problems that appeared to be related to stiffness, pain, and anxiety about his condition and the transition to high school. The patient experienced increasing chronic pain in his hips and legs with gait deterioration; consequently, he began using a wheelchair for longer distances with a shorter walking tolerance. In the same year, the ophthalmology follow-up revealed slightly elevated intraocular pressure on the right, measuring 23 mmHg, while it was normal on the left. His corneal thickness was slightly elevated to account for the borderline intraocular pressure, and an optic nerve analysis yielded normal results. At the age of 15, his height was 135 cm (below the 3rd percentile) and his weight was 40.1 kg (below the 3rd percentile). The patient exhibited elevated ferritin levels, which may be explained by the fact that patients with MBD have non-specific inflammatory markers due to osteoarthritis. A repeat MRI study showed stable findings, with progressive multilevel canal stenoses without associated cord signal abnormality that had progressed from prior imaging at the T11–T12 level.

## 6 Discussion

MBD is a rare autosomal recessive disorder that significantly affects the musculoskeletal system (1, 2). As seen in this patient, the diagnosis of MBD is typically made around the age of 4–8 years following the appearance of skeletal abnormalities such as short height and lower extremity weakness (1, 2). Diagnosis is based on mucopolysaccharides in the patient's urine, gel electrophoresis of urine oligosaccharides, and the measurement of β-galactosidase activity combined with molecular genetics testing (4, 8, 9).

Sohn et al., Sheth et al., and Ramphul et al. all have described MBD patients with scoliosis, genu valgum, kyphosis, odontoid hypoplasia, and trigger thumbs. The patient described by Sohn et al. exhibited similar radiographic findings, including anterior beaking of vertebrae, kyphoscoliosis, poorly formed acetabular roofs, and short, thick metacarpal and phalangeal bones with metaphyseal irregularity and proximal tapering (4). Similar to our patient, the literature describes mildly coarse facial features for MBD patients (4, 10). At diagnosis, our patient presented with trigger fingers. The patient described by Borgo et al. also underwent surgical correction of their trigger digits (10). Our patient was not recommended for growth hormone treatment for his short stature due to minimal benefits, consistent with literature findings (11). Nerve conduction studies were normal, and there was no evidence of neuropathy, similar to other reported cases of MBD (4).

Valgus deformities of the knee and ankle are the most common orthopedic complications seen in MBD patients (9, 10, 12). The patient described by Sohn et al. also exhibited valgus deformity of the knee and wrist and finger flexion contractures. Our patient, on the other hand, mainly experienced hamstring tightness and knee flexion contractures. During gait analysis, our patient was found to benefit from an in-shoe orthotic due to the problems brought upon by subtalar eversion worsened by knee valgus (10, 13). While the patient did not experience major problems with gait and ambulation at the time of diagnosis, his ability to walk in an unrestricted fashion had progressively deteriorated due to hip, knee, and ankle joint and ligament instability associated with the natural history of MBD (10). The patient underwent a guided growth procedure, which is in line with the recommendations in the literature (10).

Patients with MBD are known to have a predisposition for atlantoaxial instability due to a hypoplastic dens; however, our patient has not demonstrated instability clinically or radiographically (10, 12). Radiographs show our patient to have a stable kyphosis at the thoracolumbar junction, which is also well-described in the MBD patient population (10). Similar to MBD patients described in the literature, this patient has been shown on MRI to have spinal canal and lumbar exit foramina narrowing with diffuse lumbar stenosis that are asymptomatic (10, 12). While MBD patients such as the one described in this report are at risk for spinal cord compression, this is rarely reported in the literature (14).

Our patient had mild corneal clouding that was insignificant to his vision, with astigmatism over his right eye that has been previously described in Morquio patients (15, 16). Throughout his childhood and adolescence, the respiratory clinic recommended polysomnograms, as children with Morquio are at risk for sleep apnea (17, 18). These patients are at higher risk for airway obstruction due to adenotonsillar hypertrophy, which our patient experienced as a child but resolved as he transitioned to adolescence (18). MBD patients are known to be at risk for sensorineural hearing loss, for which our patient is followed up annually and has not experienced to date (15, 18, 19). Our patient also had a right-sided inguinal hernia that was surgically repaired at the age of 7, which has been reported in the literature to occur in MBD patients (15). Our patient is also well known for his mitral regurgitation and aortic systolic ejection murmur. While not uncommon, valvular pathology has been described in a smaller subset of MBD patients (9, 12, 15). The patient also had elevated ferritin levels, which is thought to be due to the fact that patients with MBD have non-specific inflammatory markers due to osteoarthritis, but this could not be confirmed with literature findings.

This case report illustrates the orthopedic challenges and subsequent treatments that a patient with MBD underwent from their diagnosis to late adolescence. In patients with MBD, guided growth should be performed proactively to maintain a normal mechanical axis and minimize mechanical imbalance. Hip containment surgery in the form of varus osteotomy and pelvic osteotomy should be considered at an early age to delay the need for arthroplasty. More case reports are needed to better understand the natural history of MBD and the various orthopedic treatment options available. Increased literature would help determine the best treatment options for these patients to reduce pain, decrease the burden of disease, and improve quality of life. To collect comprehensive, longitudinal data from more patients with MBD, authors (AC, HC, and SS) have established a Morquio-B registry housed at their institution (funded and supported by MBD patient families). This international registry will pool data from patients with MBD around the world to provide a comprehensive dataset for analysis and improving our understanding of this rare condition.

## 7 Patient perspective

Mucopolysaccharidosis type IVB (MPS IVB) affects several parts of my body, but it mainly affects my bones, making it very difficult for me to walk. My bone endings are not formed correctly, and as I get older, my tendons get looser. I was told that bone alignment from the feet up is crucial in keeping me mobile, decreasing pain in my joints, and decreasing the number of surgeries I will need later in life. I was also told that the best time to help me would be when I was growing so that my bones could be guided in the right direction rather than trying to reconstruct them after I stopped growing. By the time I was 8, I was becoming very knocked-kneed and had a lot of pain in all my joints from the waist down. The doctors watched the changes in my alignment closely until they finally needed to help me. I had screws put in my ankles, and later, screws and eight plates put in my upper tibia and lower femur at the growth plates to redirect the growth of my legs. The screws were put in on the inside of each leg to slow the growth there and allow my legs to straighten on the outside. The screws in my knees were put in and taken out a few times as I grew in order to get things right, but when I was done, my legs ended up being perfectly straight.

It took seven surgeries over 8 years to correct my legs, but I am very happy that I agreed to do it. The pain in my ankles is gone, and I have much less pain in my feet and hips and none in my knees. I also find moving a lot easier. I believe these surgeries are crucial in giving people with bone conditions a chance to have a much better life.

## Data Availability

The raw data supporting the conclusions of this article will be made available by the authors without undue reservation.
